# *socru*: typing of genome-level order and orientation around ribosomal operons in bacteria

**DOI:** 10.1099/mgen.0.000396

**Published:** 2020-06-25

**Authors:** Andrew J. Page, Emma V. Ainsworth, Gemma C. Langridge

**Affiliations:** ^1^​ Microbes in the Food Chain, Quadram Institute Bioscience, Norwich Research Park, Norwich, NR4 7UQ, UK

**Keywords:** bacteria, genome structure, rearrangement, sequencing

## Abstract

Rearrangements of large genome fragments occur in bacteria between repeat sequences and can impact on growth and gene expression. Homologous recombination resulting in inversion between indirect repeats and excision/translocation between direct repeats enables these structural changes. One form of rearrangement occurs around ribosomal operons, found in multiple copies across many bacteria, but identification of these rearrangements by sequencing requires reads of several thousand bases to span the ribosomal operons. With long-read sequencing aiding the routine generation of complete bacterial assemblies, we have developed *socru*, a typing method for the order and orientation of genome fragments between ribosomal operons. It allows for a single identifier to convey the order and orientation of genome-level structure and we have successfully applied this typing to 433 of the most common bacterial species. In a focused analysis, we observed the presence of multiple structural genotypes in nine bacterial pathogens, underscoring the importance of routinely assessing this form of variation alongside traditional single-nucleotide polymorphism (SNP) typing.

## Data Summary

All data bundled with *socru* were downloaded as complete genomes from RefSeq (accessed 26 January 2019, www.ncbi.nlm.nih.gov/refseq/). Subsets of these data were analysed in this manuscript and accession numbers are given in Table S2, available with the online version of this article.

The authors confirm that all supporting data, code and protocols have been provided within the article or through supplementary data files.

**Impact Statement**

Variation at the single-nucleotide level is a cornerstone of studies into bacterial evolution, but technologies such as long-read sequencing are now enabling us, at scale, to expand the scope to other forms of variation, such as whole-genome structure. We focused here upon inversions and translocations around repeat ribosomal operons because these operons are conserved across bacteria. We developed software called *socru* to universally type the order and orientation of bacterial genomes around these operons. The evidence presented here, that variation at the genome level was found in all nine of the pathogens we analysed in detail, provides strong impetus for genome structure to be routinely assessed alongside traditional measures of variation.

## Introduction

Bacterial genomes are dynamic entities that can undergo structural rearrangement. These rearrangements tend to occur via homologous recombination around repeat sequences, including ribosomal operons, insertion sequence (IS) elements and phage [1, 2]. Different orders and orientations of large genome fragments have been sporadically described in bacteria, including *Enterobacter *, *Salmonella *, *Staphylococcus *, *Pseudomonas* and *Listeria* [3–6]. Previously, detection of structural rearrangements has been challenging, with low-resolution methods such as restriction enzyme digestion and long-range PCR used to assay tens of strains at a time [7]. The explosion of short-read sequencing data over the past 15 years has provided the necessary resolution for identifying small changes at the DNA level, but consequently identifying structural variation at the whole-genome level has lagged behind. However, the emergence of long-read sequencing technology, which can bridge the length of long repeat sequences such as ribosomal operons, turns this situation around. As gross structural changes can impact upon growth and gene expression [8, 9], knowledge of genome structure provides a vital context in which these phenotypes can be assessed.

Currently, genome rearrangements can be identified on an ad hoc basis using synteny plots (see e.g. [10]), or through other comparative genomics methods, such as progressiveMauve [11]. progressiveMauve produces multiple genome alignments for two or more genomes that have undergone genome rearrangement, enabling these arrangements to be visualized by downstream applications such as Artemis Comparison Tool [12] or Circos [13]. However, there is no current methodology that allows complete genomes to be routinely assessed for structural rearrangement in a manner that enables swift and robust comparison within and between species, and that could be easily implemented in an analysis pipeline.

We therefore propose *socru* as a universal method for typing the order and orientation of genome fragments between ribosomal operons in complete bacterial assemblies, and present a case study examining genomes for structural variation in nine bacterial pathogens of critical importance to human health.

## Theory and implementation

### Processing of complete genomes from RefSeq

Per species, all complete genomes were downloaded from RefSeq and rRNA gene boundaries were identified using Barrnap (https://github.com/tseemann/barrnap). The nucleotide sequences (fragments) between the rRNA genes were extracted, circularized if they spanned the start/end of the assembly, and saved to individual FASTA files. Separating the fragments into separate FASTA files allowed for multiple representations of a fragment to be used, providing robustness in the method. To reduce the size of the species-specific databases, only conserved regions were kept for each fragment with a maximum length of 100 000 nucleotides. Where there were no conserved regions, the full length of the fragment was kept. A comparison of all complete genomes with full-length fragments versus conserved region fragments showed no differences in the genome structure identified.

Each fragment was compared to a database of *dnaA* nucleotide sequences using blastn (17) to identify the fragment upon which the origin of replication resided, and this was noted in the database metadata. The *dnaA* gene database was generated from complete reference genomes in RefSeq as described in Circlator (18) with similar sequences clustered using CD-hit (19) to minimize the overall file size. The termini of replication were identified by comparing each fragment against a database of *dif* nucleotide sequences using blastn, with the data drawn from Kono *et al*. (20). Of the 117 species in *socru* with an identified *dif* sequence, this sequence was located on the largest genome fragment in 102 cases. Therefore, for species with no defined *dif* sequence, the terminus was allocated to the largest genome fragment by default.

### Identification of baseline per species

**Fig. 1.**
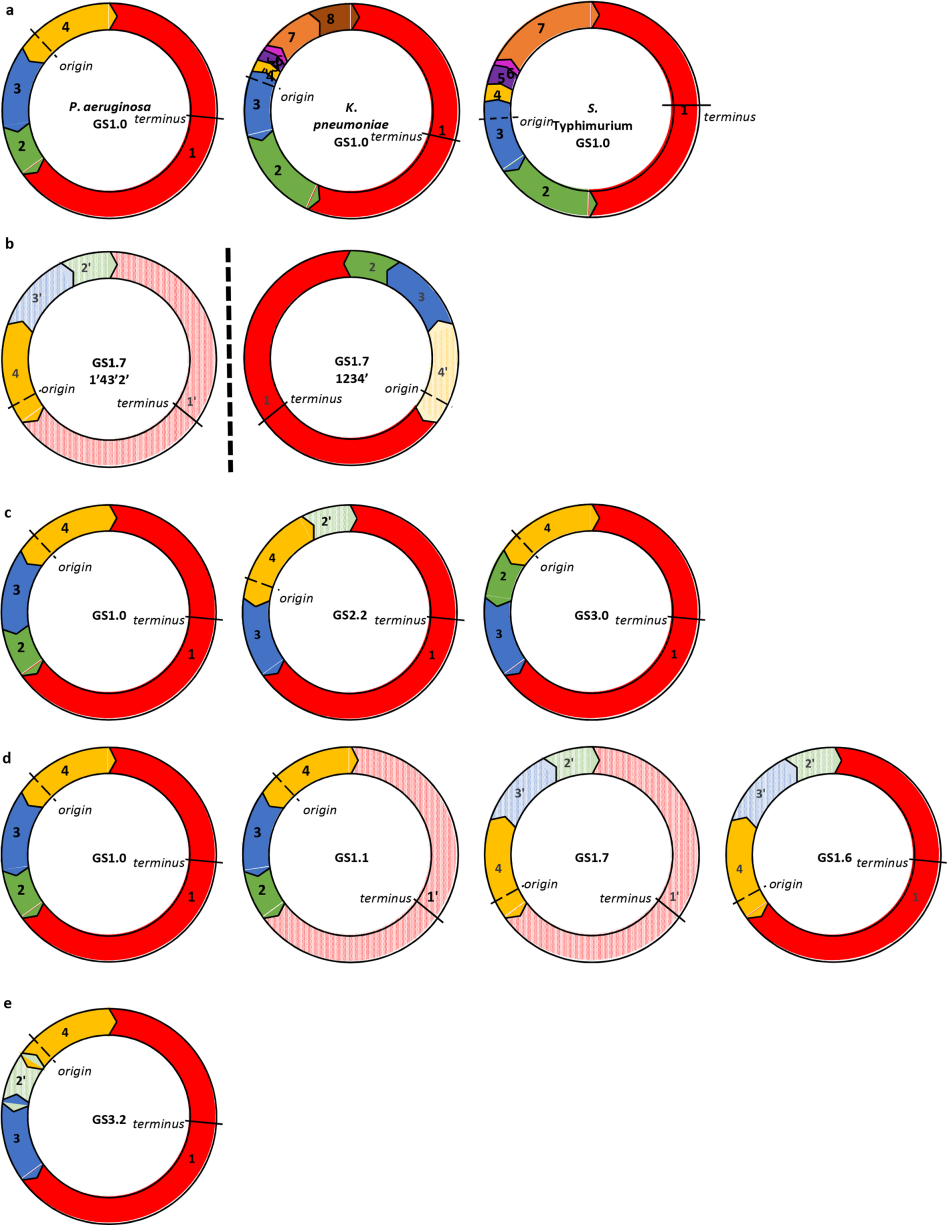
Structural genotype assignment. Coloured segments denote genome fragments, located between ribosomal operons marked as chevrons. Origin of replication (location of *dnaA*) is denoted with a dashed line and terminus (*dif* site) is denoted with a solid line. (a) Baseline references for *P. aeruginosa *, *K. pneumoniae* and *S. enterica *, indicating genome fragments running in clockwise numerical order from 1. Chevron directions indicate the orientation of ribosomal operons. The fragments harbouring the origin and terminus of replication are bordered by indirect repeats and all other fragments are bordered by direct repeats. (b) The pattern 1′43′2′ is a mirror of pattern 1234′ (flipped across the vertical dashed line). However, since *dnaA* is present on fragment 4, this fragment will always be aligned with the baseline in the forward orientation. (c) There are three valid orders in a four-fragment genome (accounting for mirroring). (d) Impact of independent inversions of fragments on orientation. GS1.0, no inversions; GS1.1, inversion of terminal fragment; GS1.7, inversion of origin fragment [represented as per mirror rule in (b)]; GS1.6, inversion of both terminal and origin fragments (as per mirror rule). (e) The assigned structural genotype is invalid – the orientation of ribosomal operons flanking fragment 2 violate the rule that operons must be oriented from the origin to the terminus of replication. This would be flagged by *socru* as a ‘red’ assignment denoting structure invalidity, which is indicative of potential misassembly.

A complete reference genome was required to provide a baseline order and orientation for each species. The complete reference genome with the lowest numerical GCF accession number in RefSeq was chosen as the baseline in each case (Fig. 1a). *socru* was written for circular genomes but can be utilized for and is populated with linear genomes in addition. Here, we discuss circular genomes in particular, but the same general principles apply to linear genomes. Genome fragments were labelled numerically from 1, beginning with the largest fragment and working in a clockwise fashion around the chromosome. Genome structures were represented using these fragment numbers relative to the baseline, with inverted orientations denoted with prime (′).

### Structural genotype assignment

For all complete RefSeq genomes in a species, each unique pattern was given a unique genome structure (GS) identifier. This facilitates comparison of the overall structural variation in a population. A database for each species contains a tab delimited table of these patterns. The genome structure identifier takes the form GSX.Y (e.g. GS1.6), where X uniquely denotes the order of the fragments and Y denotes the orientation of the fragments (i.e. whether inverted or not, relative to the baseline). For circular genomes, the number of valid orders and orientations is determined by the number of genome fragments, which is explained in detail below. For *n* fragments, taking mirrored structures into account, the maximum number of theoretically possible structural genotypes is 2×[(*n*−1)!].

### Genome order

For the genome order to be valid and accepted by *socru*, the ribosomal repeat sequences must be oriented in the forward direction from the origin of replication towards the terminus (Fig. 1a). For each new GS assignment, the orientation of the whole genome is always relative to the baseline fragment with *dnaA* in the forward direction, to provide consistency in the patterns. This also prevents issues of mirrored structures (e.g. Fig. 1b). For *n* fragments, the total number of genome orders, regardless of the orientation of the terminus and origin fragments, is (*n*−1)!]/2. As an example, *Pseudomonas aeruginosa* has four genome fragments, corresponding to three genome orders (Fig. 1c).

### Genome orientation

The orientation is an integer representation of the orientation of the fragments in binary in reverse order (as this allows for variability in the number of fragments), where 0 indicates the same direction and 1 indicates reverse direction relative to the baseline. For example, *P. aeruginosa* baseline structure 1234=>0000, which is represented as GS1.0, while structure 143′2′= >0111 and is represented as GS1.7 (Fig. 1d, Table S1). In each unique genome order there are four valid orientations, which correlate to the four possible combinations of the orientations of the origin and terminus fragments. This is because these fragments are flanked by inverted repeats of the ribosomal operon; all other fragments are flanked by direct repeats.

### Pattern validity

Patterns were accepted if they contained the same number of fragments as the baseline, each occurring exactly once, and the rRNA operons were orientated in a biologically valid manner, i.e. going from the origin of replication to the terminus of replication. When using *socru* with a new query genome, readouts from these checks provide an indication of the validity of the structure and hence also aid in spotting misassemblies (e.g. Fig. 1e).

### Software databases

*socru* is bundled with a set of prepopulated databases covering 7401 genomes across 433 species. These represent the species with three or more complete reference assemblies available in RefSeq (accessed 26 January 2019), and where the reference sequence contained three or more rRNA operons. The databases are openly available on Github.com, which allows for community curation and enhancements.

### Software usage and availability

Given a FASTA file of a complete bacterial assembly, *socru* utilizes a database (prebundled or user provided) to identify the structural genotype. First the location of the rRNA genes is identified with Barrnap. Using blastn, the sequence similarity is calculated between the user provided assembly and the reference genome fragments. The blast results are filtered (user definable, defaulting to: evalue 0.000001, minimum bit score 100, minimum alignment length of 100 bases), and the match with the highest bit score is used to identify the fragment number and the orientation. The order and orientation of the fragments are looked up in the bundled database of GS numbers. Novel orders are given a GS number of 0, which the researcher can evaluate for biologically probability. The output consists of the input file name, the GS identifier, a red/amber/green quality indication of structure validity and genome structure pattern. Red denotes invalid structure, while amber indicates that the structure is valid but requires user confirmation of a novel genome order. Users are encouraged to add novel, valid structural assignments to the relevant *socru* species database. Green denotes assignment of a structural genotype matching one already present in the species database. The software requires less than 250 MB of RAM to run and takes about 20 s to process a single 5 Mbase assembly on a standard laptop. *socru* is available under the open source GNU GPL 3 licence from https://github.com/quadram-institute-bioscience/socru. The software is written in Python 3, validated using unit tests and packaged for Conda, Galaxy, Docker and Pip for easy installation.

### Case study: ESKAPE pathogens

We assessed structural variation in all available complete genomes for the ESKAPE pathogens (* Enterococcus faecium *, *Staphylococcus aureus *, *Klebsiella pneumoniae *, *Acinetobacter baumannii *, *P. aeruginosa *, and *Enterobacter* spp.) as well as *Escherichia coli *, *Salmonella enterica* and *Listeria monocytogenes *. Structural genotypes defining the order and orientation of genome fragments around ribosomal operons were assigned with *socru* based upon the comparison of each assembly to a species-specific baseline (Table S2) and the Methods section).

All of the bacterial species analysed displayed at least 5 structural genotypes; *S. enterica* was the most diverse with almost 30 (Table 1). The dominant type in all but two cases was the baseline structure, termed GS1.0. For *P. aeruginosa *, the baseline structure of GS1.0 came from PAO1, but our results showed that 88 % (*n*=151) of all *P. aeruginosa* genomes harboured fragment 1 in the inverted orientation, designated GS1.1 (Fig. 1d). Indeed, only eight genomes reflected the same order and orientation as PAO1. That PAO1 harbours an inversion was documented in the original sequencing paper [14], but this analysis demonstrates how rare this genome structure is in *P. aeruginosa *.

**Table 1.** Structural variation in bacterial pathogens

Pathogen (baseline)	Baseline no. of fragments (total possible combinations)	No. of complete RefSeq genomes	No. of observed arrangements	Main GS type	% with main GS type	No. of likely misassemblies in database
* E. faecium* (DO)	6 (240)	116	5	GS1.0	69 %	12
* S. aureus* (RF122)	5 (48)	408	10	GS2.0*	59 %	29
* K. pneumoniae* (NTUH-K2044)	8 (10 080)	350	7	GS1.0	98 %	21
* A. baumannii* (ACICU)	6 (240)	148	6	GS1.0	72 %	6
* P. aeruginosa* (PAO1)	4 (12)	185	6	GS1.1	88%	13
* Enterobacter* spp. (multiple)	7 or 8 (up to 10 080)	88	5	GS1.0	63 %	16
* E. coli* (K12 MG1655)	7(1440)	838	13	GS1.0	90 %	62
* L. monocytogenes* (4b_F2365)	6(240)	176	5	GS1.0	91 %	56
* S. enterica* (LT2)	7(1440)	726	29	GS1.0	79 %	25
Non-*S*. Typhi		*607*	*15*	GS1.0	94 %	18
*S*. Typhi		*119*	*17*	GS2.67	66 %	7

Baseline genome accessions: DO GCF_000174395.2, RF122 GCF_000009005.1, NTUH-K2044 GCF_000009885.1, ACICU GCF_000018445.1, PAO1 GCF_000006765.1, K12 MG1655 GCF_000005845.2, 4b_F2365 GCF_000008285.1, LT2 GCF_000006945.2. *Enterobacter* spp. comprised the following species and baselines: seven fragments, *Enterobacter* sp. 638 GCF_000016325.1; eight fragments, *E. asburiae* L1 GCF_000632395.1, *E. cloacae* ATCC13047 GCF_000025565.1, *E. hormaechei* ECNIH3 GCF_000750225.1 and *E. roggenkampii* 35 734 GCF_000807415.2.

*S. aureus GS2.0 harbours six fragments, whereas GS1.0 (36 %) harbours five. *S. enterica* subdivided to show structural genotypes found in *S. enterica* subspecies *enterica* serovar Typhi (*S*. Typhi) versus the remainder of *S. enterica*.

Where GS1.0 was the dominant structural genotype, its frequency varied appreciably between species (Table 1). *socru* typing of *K. pneumoniae* complete genomes provided strong support for structural conservation in this species [15], with 98 % (*n*=326) displaying GS1.0. Conversely, in *Enterobacter* spp., *E. faecium* and *A. baumannii *, a lower frequency of GS1.0 was observed (63–72 %). As the first complete sequenced reference genome for each species was used as the baseline for our structural genotyping, the low GS1.0 frequencies demonstrate empirically that ‘first’ genomes, though often an important laboratory strain or of clinical importance, are not always representative of the structure of the species as a whole.

In *S. aureus *, it has been noted that isolates may contain five or six ribosomal operons, with five copies being postulated as an adaptation to antibiotic pressure in a hospital environment [16]. Our data demonstrate that it is consistently the same ribosomal operon that is absent in five-copy complete genomes (*n*=138, Table S2) one that is approximately 300 bp away from the next operon, with no obvious genetic features in between. However, six-copy genomes are numerous in RefSeq (*n*=227, designated GS2.0), suggesting that there is some selective pressure to maintain a sixth copy.

Since *S. enterica* harboured the greatest number of structural genotypes, we looked more closely at how these were distributed across the 726 available genomes. It was striking to note that a single serovar (*S*. Typhi) was responsible for over half of the observed structures, i.e. more than the rest of the species combined. Structural variation in *S*. Typhi, the causal agent of typhoid fever, has been associated with persistence in the human host [7]. A link with persistence has also been demonstrated in *S. aureus* [17]. Persistence in bacterial populations is typified by reduced growth rate and antimicrobial tolerance [18], both of which may be explained by structural variation, indicating a fruitful direction for future research.

### Identification of misassemblies

In addition to the biological significance of structural variation, our study also highlights an important issue regarding the quality of some complete genome assemblies. Valid structures were assigned based upon certain rules governing operon direction and fragment inversion, excision and translocation (Fig. S1) . Cases where fragments were missing or repeated, or operon directions violated the origin to terminus of replication order, were deemed possible misassemblies. An analysis of all the ESKAPE genomes (*n*=1295) found that 6.76 % (*n*=88) were biologically invalid and likely the result of misassemblies. As such, *socru* can also be used to identify large-scale misassemblies and therefore provide a useful quality control step in high-throughput bacterial assembly pipelines.

## Data Bibliography

Complete RefSeq genomes for bacterial species are bundled with *socru* that have a) at least 3 ribsosomal operons and b) have at least 3 RefSeq genomes for a given species. https://www.ncbi.nlm.nih.gov/refseq/ accessed 2019-01-26. Accession numbers for 3042 complete RefSeq genomes of ESKAPE pathogens, *E. coli*, *L. monocytogenes* and *S. enterica* from https://www.ncbi.nlm.nih.gov/refseq/ are given in Table S2.

## Supplementary Data

Supplementary material 1

Supplementary material 2
