# Supplementary figures and images for: socru: typing of genome-level order and orientation around ribosomal operons in bacteria
Source: Microb Genom. 2020 Jun 25;6(7):mgen000396. doi: 10.1099/mgen.0.000396 (PMC7478630; doi:10.1099/mgen.0.000396)

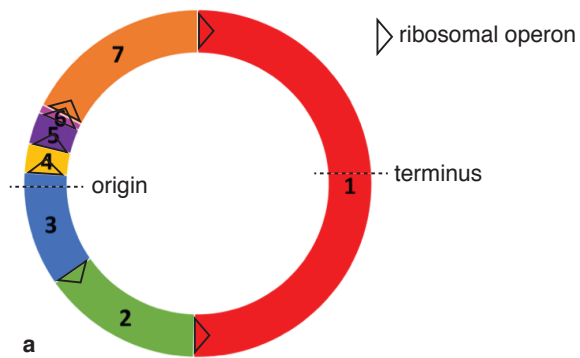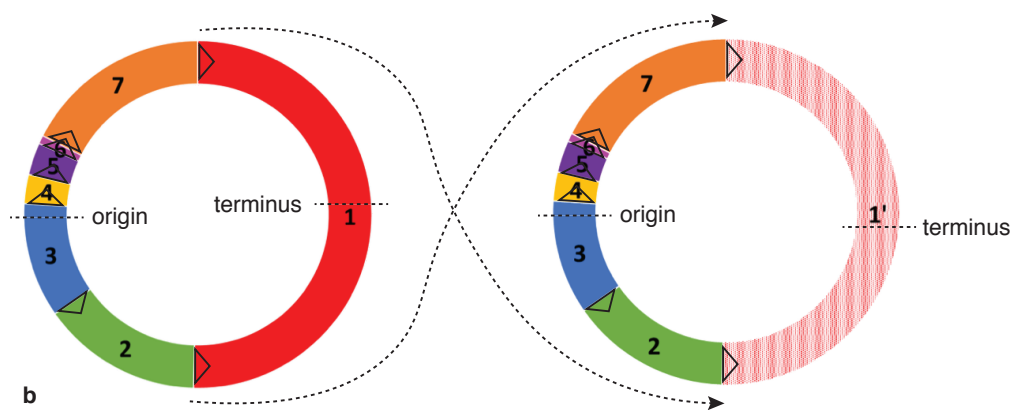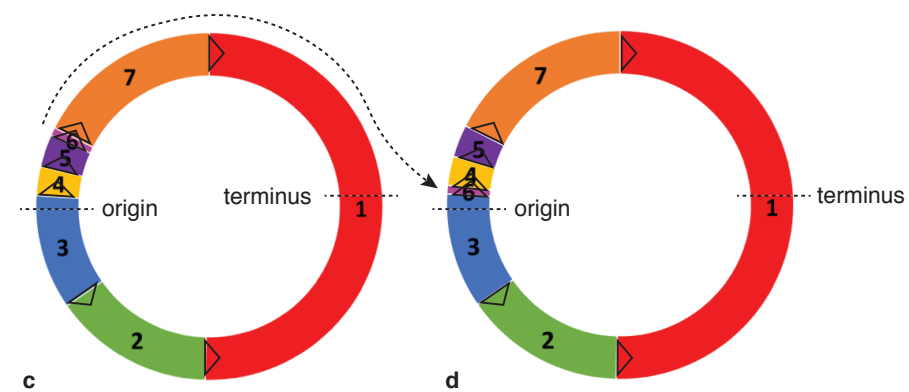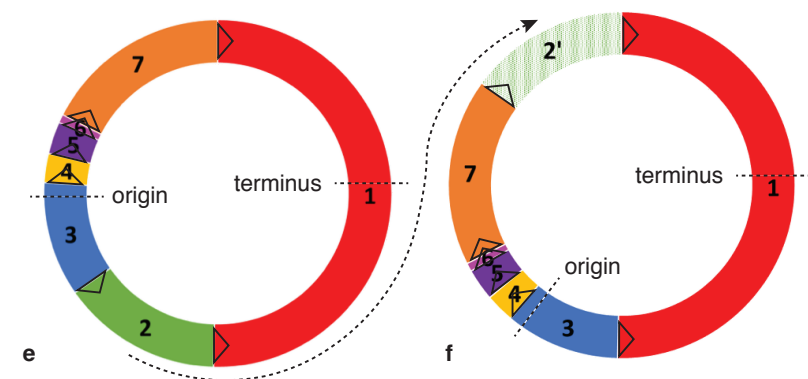

Supplement: Supplementary material 1 [file mgen-6-396-s001.pdf]
